# Comparison of three different serum-free light-chain assays—implications on diagnostic and therapeutic monitoring of multiple myeloma

**DOI:** 10.1038/s41408-019-0267-8

**Published:** 2020-01-09

**Authors:** Aneta Schieferdecker, Sebastian Hörber, Monika Ums, Britta Besemer, Carsten Bokemeyer, Andreas Peter, Katja Weisel

**Affiliations:** 1 Department of Oncology and Hematology, BMT with Department of Pneumology, Hubertus Wald Tumorzentrum, University Medical Center Hamburg-Eppendorf, Hamburg, Germany; 2 Institute for Clinical Chemistry and Pathobiochemistry, Department for Diagnostic Laboratory Medicine, University Hospital Tuebingen, Tuebingen, Germany; 3 Institute for Diabetes Research and Metabolic Diseases of the Helmholtz Center Munich at the University of Tuebingen, Tuebingen, Germany; 4 German Center for Diabetes Research (DZD), München-Neuherberg, Germany; 5 Center of Clinical Trials (ZKS) Tübingen, University Hospital of Tuebingen, Tuebingen, Germany; 6 Department of Hematology, Oncology, Immunology and Rheumatology, University Hospital of Tuebingen, Tuebingen, Germany

**Keywords:** Myeloma, Myeloma

## Abstract

The measurement of serum-free light chains (FLC) is standard of care in the diagnosis and management of multiple myeloma (MM). The revised international myeloma working group (IMWG) implemented the involved FLC/noninvolved FLC (iFLC/niFLC) ratio as a biomarker for MM requiring treatment. Recently, a new definition of high-risk smoldering MM (SMM) including iFLC/niFLC ratio was published. These recommendations were solely based on a single assay method (Freelite assay). Today, two additional assays, N Latex FLC and ELISA-based Sebia FLC, are available. Here, we report on a single-center-study comparing results of all three different assays for FLC correlation and its potential implications for diagnostic and clinical use. In total, 187 samples from 47 MM patients were examined, and determination of FLC was performed. Comparison analyses showed similar FLC results for Sebia FLC and N Latex FLC assay with markedly lower absolute values for κ/λ ratio compared with Freelite. Values of λ FLC exhibited high variability. The ratio of iFLC/niFLC showed significant discrepancies among these assays. Our data demonstrate that the three available assays may result in markedly discrepant results, and should not be used interchangeably to monitor patients. Furthermore, modifications of the assay-specific diagnostic (iFLC/niFLC) thresholds for SMM and MM are recommended.

## Introduction

Serum-free light chains (FLC) are important biomarkers for the diagnosis and management of smoldering multiple myeloma (SMM), multiple myeloma (MM), and other plasma cell disorders, such as monoclonal gammopathy of undetermined significance (MGUS), light-chain amyloidosis (AL-amyloidosis), and light-chain deposition disease (LCDD)^1–6^.

In MM patients, the determination of FLC is used in the initial diagnostic assessment and during follow-up monitoring. In response evaluation, determination of stringent complete response (sCR) is defined by complete response (negative immunofixation in serum and urine, plasma cells in the bone marrow < 5%) with normalized kappa/lambda ratio (κ/λ ratio)^3^. In the absence of a measurable serum and urine M-protein, response assessment is based on the percentage decrease of difference between involved and noninvolved FLC (partial response is defined as dFLC decrease > 50%)^7^. Furthermore, in LCDD and AL-amyloidosis, response assessment relies on absolute and percentage decrease of difference between involved and noninvolved FLC (partial response is defined as dFLC decrease > 50% and very good partial response as dFLC < 40 mg/l)^4–6^.

The importance of FLC measurement was recently highlighted by introduction of a new definition of MM disease. According to the recommendation of the International Myeloma Working Group (IMWG), the FLC ratio of involved and noninvolved FLC (iFLC/niFLC ratio) ≥ 100, with concentration of involved FLC ≥ 100 mg/l is sufficient to differentiate between SMM and MM requiring treatment^8,9^. Even more recently, new criteria for high-risk SMM were defined^10^. The 2/20/20 rule with serum M-protein level > 2 g/l, FLC ratio > 20, and bone marrow infiltration > 20% identifies SMM patients with a progression rate of 46% within 2 years and who might benefit from early treatment intervention.

Since the implementation of FLC measurement in diagnostic evaluation of MM, only a single assay method based on polyclonal antibodies (Freelite) was accessible for measurement of FLC^11^. Subsequently, two additional assays were developed: the N Latex FLC assay, which is based on monoclonal antibodies, introduced in 2011^12^, and the Sebia FLC assay, launched in 2018, which is an ELISA-based assay using polyclonal antibodies^13^. To date, data and recommendations out of clinical trials were exclusively relying on the results obtained with the Freelite assay.

When using FLC assays, different potential analytical limitations have been described and have to be considered, including antigen excess, underestimation, overestimation, lot-to-lot variation and nonlinearity^1^. The use of polyclonal antibodies enables broader light-chain recognition, but may also result in higher lot-to-lot variation. Monoclonal antibodies, however, may increase the risk of antigen excess and some paraproteins may escape detection.

So far, only a limited number of comparison studies between the different assays exist. Previous comparisons between N Latex and Freelite assay^14–19^ and between Sebia FLC and Freelite assay^13,20,21^ have shown discrepancies in determination of light-chain values and κ/λ ratio. In comparison analyses between Freelite and N Latex FLC, and between Freelite and Sebia FLC, absolute FLC concentrations measured by Sebia FLC or N Latex FLC were lower compared with Freelite. Two recently published studies showed that the diagnostic thresholds for iFLC/niFLC ratio ≥ 100 proposed for Freelite do not apply to N Latex FLC and Sebia FLC. New thresholds for N Latex FLC (iFLC/niFLC ratio ≥ 30) and for Sebia FLC (iFLC/niFLC ratio ≥ 16) were proposed^14,20^.

This is very important clinically because, due to different results of FLC assays, patients may not be detected as MM patients requiring treatment, responses might not be evaluated consistently, and treatment decisions may be “test-dependent” and not truly disease dependent.

Here, we report for the first time on a comparison of all three FLC assays (Freelite, N Latex FLC, and Sebia FLC) on the same patient samples in calculation of absolute FLC values, κ/λ ratios, and iFLC/niFLC ratios. The results will be discussed in terms of clinical relevance and consequences.

## Materials and methods

### Study design

Fifty-two patients were included into the trial from April 2016 to March 2017. The results of all three FLC assays were available from 47 patients with a total number of 187 samples. Fresh serum samples were taken at the beginning of the study and at each follow-up visit (median 5, range 1–7). Written informed consent was obtained from all participants at the beginning of the study. The study was approved by regional authorities according to declaration of Helsinki (IRB number 052/2016B02).

### Sample collection and FLC assays

Blood samples were collected via direct venipuncture following standard operating procedures at the beginning of the study and after a maximum of six follow-up visits. Serum samples were stored at 4–8 °C for complete coagulation and were subsequently centrifuged. Supernatants were removed and transferred into at least four aliquots, and stored at 4 °C or were immediately frozen and stored at −20 °C.

Determination of FLC (κ, λ, and κ/λ ratio) was conducted with three different assays. N Latex FLC reagents (Siemens Healthineers, Eschborn, Germany) and Freelite reagents (The Binding Site (TBS), Birmingham, UK) were used on a Siemens BN II nephelometer with fresh serum aliquots. Sebia FLC, based on solid-phase sandwich enzyme-linked immunosorbent assay (ELISA; Sebia, Evry, France), was performed manually using frozen aliquots. Assay-specific reference ranges for κ and λ FLC and κ/λ ratio are shown according to the manufacturer in Supplementary Table 1. Linearity and precision analyses of Sebia FLC were performed. The analytical performance of N Latex FLC and Freelite measured by the BN II nephelometer was extensively examined in previous studies^15,22,23^.

### Statistical analysis

Comparison analyses between FLC assays were performed using Passing–Bablok regression analysis. The Spearman rank correlation coefficient (*r*_s_) was determined to evaluate correlation between methods. Correlation was graded according to suggestions proposed by Evans:^24^ <0.20 indicates a very weak; 0.20–0.39 a weak; 0.40–0.59 a moderate; 0.60–0.79 a strong, and >0.80 a very strong correlation. Agreement of different FLC assay results was compared using Bland–Altman plots. Concordance was obtained and calculated using contingency tables, and Cohens Kappa coefficients were calculated for interobserver agreement. The results of Cohens Kappa were evaluated as proposed by Altman:^25^ <0.2 indicates a poor, 0.21–0.40 a fair, 0.41–0.60 a moderate, 0.61–0.80 a good, and >0.81 a very good concordance. Passing–Bablok regression analysis was conducted with MedCalc software (Version 18.3; MedCalc Software, Ostend, Belgium). Bland–Altman analyses and determination of Spearman rank correlation coefficient were performed using GraphPad Prism software (GraphPad Software, San Diego, USA). Contingency tables were performed using Microsoft Excel (Microsoft Corporation, Redmond, USA), and Cohens Kappa coefficients were calculated using JMP software (version 14; SAS Institute, Cary, USA).

## Results

### Performance of Sebia FLC assay

First, the analytical performance of the novel Sebia FLC assay was investigated. Sebia FLC shows high linearity for measurements of κ FLC (*R*^2^ = 0.97) and λ FLC (*R*^2^ = 0.99; Supplementary Table 2) and revealed good intraassay precision for determination of κ FLC (7.7% at 5.6 mg/l and 6.1% at 27.3 mg/l) and λ FLC (15.7% at 4.0 mg/l and 10.4% at 29.1 mg/l). Interassay precision was between 12.0 and 23.1% (κ FLC) and 9.2 and 12.8% (λ FLC) for κ and λ FLC concentrations of 5–90 mg/l, respectively (Supplementary Tables 3–5). Antigen excess was never observed with the Sebia FLC during the entire study with values up to 6093 mg/l (κ FLC) and 1385 mg/l (λ FLC). Following the manufacturer’s instructions, 61 of 374 measurements (κ and λ FLC) had to be repeated because primary results were outside the initial measuring ranges.

### Study population

In total, 47 patients were included in the final analysis with a total number of 187 samples. Among them, 31 patients had newly diagnosed and 16 patients had relapsed and/or refractory multiple myeloma. Thirty-three patients showed MM with clonal secretion of a complete immunoglobulin (18 with κ FLC and 15 with λ FLC), eight patients had light-chain multiple myeloma (LCMM) (3 with κ FLC and 5 with λ FLC). Six patients were classified as SMM (five with κ FLC, one with λ FLC). Median age was 63 years. Patients’ characteristics are shown in Table 1.

**Table 1:** Patients’ characteristics.

Characteristic (*n* = 47)	No. (%)
*Gender*	
Female	19 (40%)
Male	28 (60%)
*Age (years)*	40–82 (median 63)
≤65	27 (57%)
>65	20 (43%)
*Type*	
IgG	31 (66%)
IgA	8 (17%)
Light chain	8 (17%)
*Diagnosis*	
Smoldering myeloma	6 (13%)
Multiple myeloma	41 (87%)
*Setting*	
Newly diagnosed	31 (66%)
Refractory/relapsed	16 (34%)

### Comparison of Freelite, N Latex FLC, and Sebia FLC

**Fig. 1:**
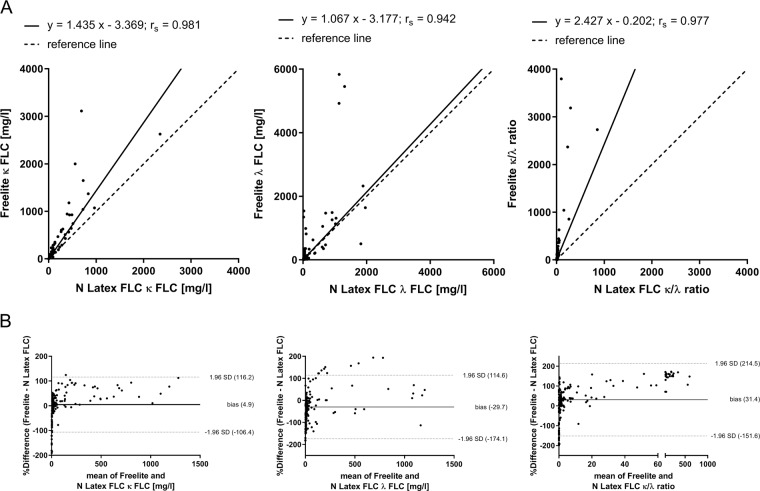
Comparison of N Latex FLC with Freelite in the determination of FLC. Passing–Bablok (**a**) and Bland–Altman (**b**) analyses were performed using 187 serum samples from patients with newly diagnosed or relapsed multiple myeloma (MM, *n* = 33), light-chain multiple myeloma (LCMM, *n* = 8) or smoldering multiple myeloma (SMM, *n* = 6). Shown are the results for the determination of κ and λ FLC and κ/λ ratio determined by N Latex FLC and Freelite. Bland–Altman plots reveal agreement between N Latex FLC and Freelite. A positive bias indicates higher values for the determination of FLC by Freelite compared with N Latex FLC. For a better representation of FLC results, two samples with extreme κ FLC results were not shown (sample 1: κ FLC results of Freelite: 14500 mg/l, N Latex FLC: 11200 mg/l, Sebia FLC: 3456 mg/l; sample 2: κ FLC results of Freelite: 31800 mg/l, N Latex FLC: 5880 mg/l, Sebia FLC: 6093 mg/l).

In the first part of the clinical study, a comparitive analysis of the most widely used FLC assays, Freelite and N Latex FLC, was performed using fresh serum samples. The results of κ FLC (*r*_s_ = 0.981, *p* < 0.0001), λ FLC (*r*_s_ = 0.942, *p* < 0.0001), and κ/λ ratio (*r*_s_ = 0.977, *p* < 0.0001) determination showed a strong correlation between N Latex FLC and Freelite (Fig. 1a). Bland–Altman plots were performed to evaluate differences between N Latex FLC and Freelite. The results for κ FLC (bias = 4.9%), λ FLC (bias = −29.7%), and κ/λ ratio (bias = 31.4%) are shown in Fig. 1b. To evaluate the agreement between N Latex FLC and Freelite results, contingency analyses were performed. These analyses showed a very good concordance for κ FLC (Kappa coefficient 0.81) and a moderate concordance for λ FLC (Kappa coefficient 0.56) between N Latex FLC and Freelite (Table 2).

**Table 2:** Concordance of FLC measurements.

(A) Concordance of κ FLC								
κ FLC	N Latex FLC		κ FLC	Sebia FLC		κ FLC	Sebia FLC	
Freelite	Normal	Abnormal	Freelite	Normal	Abnormal	N Latex FLC	Normal	Abnormal
Normal	70 (37%)	11 (6%)	Normal	64 (34%)	12 (7%)	Normal	65 (35%)	16 (9%)
Abnormal	6 (3%)	100 (54%)	Abnormal	13 (7%)	98 (52%)	Abnormal	12 (6%)	94 (50%)
Cohens Kappa coefficient: 0.81			Cohens Kappa coefficient: 0.72			Cohens Kappa coefficient: 0.69		
*(B) Concordance of λ FLC*								
**λ FLC**	**N Latex FLC**		**λ FLC**	**Sebia FLC**		**λ FLC**	**Sebia FLC**	
Freelite	Normal	Abnormal	Freelite	Normal	Abnormal	N Latex FLC	Normal	Abnormal
Normal	50 (27%)	22 (12%)	Normal	44 (23%)	28 (15%)	Normal	40 (21%)	26 (14%)
Abnormal	16 (8%)	99 (53%)	Abnormal	18 (10%)	97 (52%)	Abnormal	22 (12%)	99 (53%)
Cohens Kappa coefficient: 0.56			Cohens Kappa coefficient: 0.47			Cohens Kappa coefficient: 0.43		
*(C) Concordance of κ/λ ratio*								
**κ/λ ratio**	**N Latex FLC**		**κ/λ ratio**	**Sebia FLC**		**κ/λ ratio**	**Sebia FLC**	
Freelite	Normal	Abnormal	Freelite	Normal	Abnormal	N Latex FLC	Normal	Abnormal
Normal	42 (22%)	11 (6%)	Normal	34 (18%)	19 (10%)	Normal	39 (21%)	13 (7%)
Abnormal	10 (5%)	124 (67%)	Abnormal	17 (9%)	117 (63%)	Abnormal	12 (6%)	123 (66%)
Cohens Kappa coefficient: 0.72			Cohens Kappa coefficient: 0.52			Cohens Kappa coefficient: 0.66		

Assay-specific reference ranges were used for the classification of a normal (within reference range) and abnormal (outside reference range) FLC. Determination of κ, λ, and κ/λ ratio concordances were performed using 187 serum samples

**Fig. 2:**
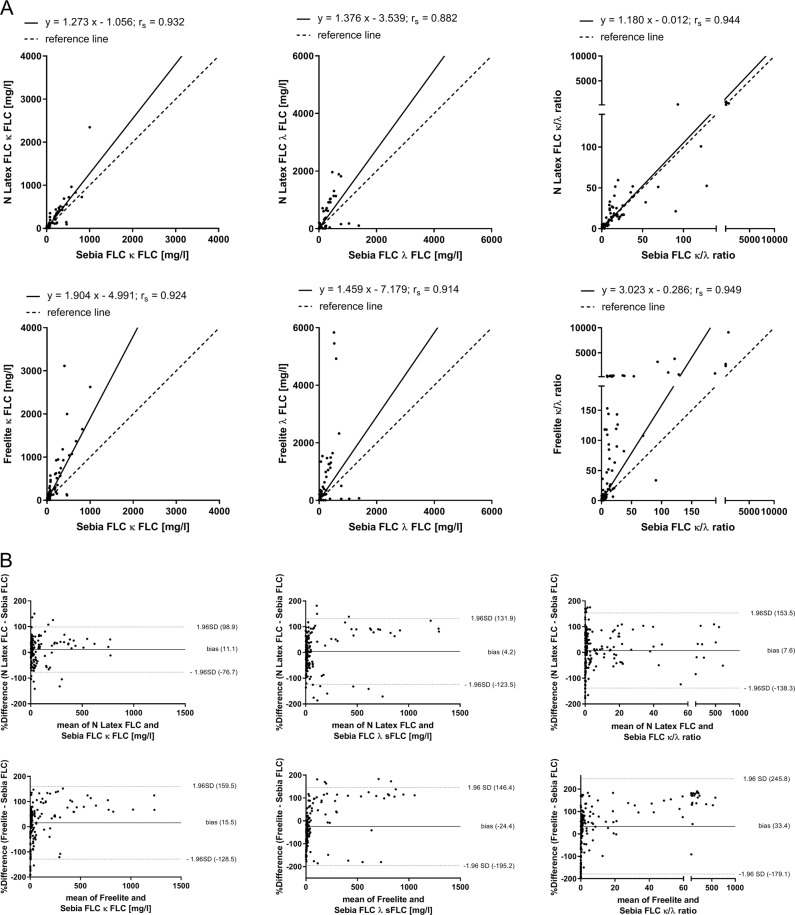
Comparison of Sebia FLC with N Latex FLC and Freelite in the determination of FLC. The results of κ and λ FLC and κ/λ ratio determination by Sebia FLC are compared with N Latex FLC and Freelite results using 187 serum samples from patients with newly diagnosed or relapsed multiple myeloma (MM, *n* = 33), light-chain multiple myeloma (LCMM, *n* = 8), or smoldering multiple myeloma (SMM, *n* = 6). Shown are the results of Passing–Bablok (**a**) and Bland–Altman (**b**) analyses. Bland–Altman plots indicate agreement between FLC assays. A positive bias indicates higher values for determination of FLC by Freelite or N Latex FLC compared with Sebia FLC. For a better representation of FLC results, four samples with extreme κ FLC results or κ/λ ratios were not shown (sample 1: κ FLC results of Freelite: 14,500 mg/l, N Latex FLC: 11,200 mg/l, Sebia FLC: 3456 mg/l; sample 2: κ FLC results of Freelite: 31,800 mg/l, N Latex FLC: 5880 mg/l, Sebia FLC: 6093 mg/l; sample 3: κ/λ ratios of Freelite: 62,281, N Latex FLC: 727, Sebia FLC: 214; sample 4: κ/λ ratios of Freelite: 27146, N Latex FLC: 605, Sebia FLC: 406).

In the second part of the study, the results of Sebia FLC, the novel ELISA-based FLC assay from Sebia, were compared with previously evaluated results of N Latex FLC and Freelite. κ FLC measurement results of Sebia FLC showed a strong correlation with N Latex FLC (*r*_s_ = 0.932, *p* < 0.0001) and Freelite (*r*_s_ = 0.924, *p* < 0.0001; Fig. 2a). Furthermore, a strong correlation was found for determination of λ FLC between Sebia FLC and N Latex FLC (*r*_s_ = 0.882, *p* < 0.0001) and Sebia FLC and Freelite (*r*_s_ = 0.914, *p* < 0.0001). κ/λ ratios determined by Sebia FLC exhibited a strong correlation with N Latex FLC (*r*_s_ = 0.944, *p* < 0.0001) and Freelite (*r*_s_ = 0.949, *p* < 0.0001). Differences between Sebia FLC and N Latex FLC results were calculated for κ FLC (bias = 11.1%), λ FLC (bias = 4.2%), and κ/λ ratios (bias = 7.6%; Fig. 2b). Furthermore, differences between Sebia FLC and Freelite results were also calculated for κ FLC (bias = 15.5%), λ FLC (bias = −24.4%), and κ/λ ratios (bias = 33.4%). Finally, concordances between Sebia FLC and both other FLC assays were determined. Good concordance was demonstrated between Sebia FLC and N Latex FLC (Kappa coefficient 0.69) and between Sebia FLC and Freelite (Kappa coefficient 0.72) for κ FLC determination. Considering determination of λ FLC, only a moderate concordance was calculated between Sebia FLC and N Latex FLC (Kappa coefficient 0.43) and Sebia FLC and Freelite (Kappa coefficient 0.47; Table 2).

### Evaluation of k/λ ratio and involved FLC/noninvolved FLC (iFLC/niFLC) ratio

To evaluate the concordance on the determination of an abnormal κ/λ ratio (outside the assay-specific reference range), contingency analyses were performed (Table 2c). N Latex FLC revealed a good concordance for κ/λ ratio with Freelite (Kappa coefficient 0.72) and Sebia FLC (Kappa coefficient 0.66), and Sebia FLC showed a moderate concordance with Freelite (Kappa coefficient 0.52).

The IMWG recommends an iFLC/niFLC ratio ≥ 100, when using the Freelite assay, as a diagnostic criteria for determination of MM requiring treatment. Therefore, the concordance between the results of Sebia FLC or N Latex FLC and the results of Freelite were compared (Table 3). Using Freelite, 18 of 42 samples showed an iFLC/niFLC ratio ≥ 100. N Latex FLC revealed an iFLC/niFLC ratio ≥ 100 for 10 samples, and Sebia FLC revealed 9 samples with an iFLC/niFLC ratio ≥ 100 out of the 42 samples. Furthermore, we expanded the analysis on the iFLC/niFLC ratio > 20, which is used for the definition of high-risk SMM. Again, the number of samples with an iFLC/niFLC ratio > 20 at baseline were compared between all three assays (Table 3). Twenty-nine samples showed an iFLC/niFLC ratio > 20 using Freelite, 20 and 22 samples were identified by N Latex FLC and Sebia FLC, respectively.

**Table 3:** Concordance of iFLC/niFLC.

(A) Comparison of Freelite thresholds for MM requiring treatment (iFLC/niFLC ratio ≥ 100) and high-risk SMM (iFLC/niFLC ratio > 20) with N Latex FLC and Sebia FLC.				
iFLC/niFLC	N Latex FLC		Sebia FLC	
Freelite	<100	≥100	<100	≥100
<100	23 (56%)	1 (2%)	23 (55%)	1 (2%)
≥100	9 (21%)	9 (21%)	10 (24%)	8 (19%)
	Cohens Kappa coefficient: 0.49		Cohens Kappa coefficient: 0.43	
Freelite	≤20	>20	≤20	>20
≤20	13 (31%)	0 (0%)	13 (31%)	0 (0%)
>20	9 (21%)	20 (48%)	7 (17%)	22 (52%)
	Cohens Kappa coefficient: 0.58		Cohens Kappa coefficient: 0.66	

(B) Use of assay-specific thresholds for MM requiring treatment (iFLC/niFLC ratio ≥ 100/ ≥ 50/ ≥ 20) and high-risk SMM (iFLC/niFLC ratio > 20/ > 8/ > 8).				
iFLC/niFLC	N Latex FLC		Sebia FLC	
Freelite	<50	≥50	≤20	>20
<100	23 (55%)	1 (2%)	18 (43%)	6 (14%)
≥100	5 (12%)	13 (31%)	2 (5%)	16 (38%)
	Cohens Kappa coefficient: 0.70		Cohens Kappa coefficient: 0.62	

iFLC/niFLC	N Latex FLC iFLC/niFLC		Sebia FLC iFLC/niFLC	
Freelite	≤8	>8	≤8	>8
≤20	12 (29%)	1 (2%)	12 (29%)	1 (2%)
>20	2 (5%)	27 (64%)	2 (5%)	27 (64%)
	Cohens Kappa coefficient: 0.84		Cohens Kappa coefficient: 0.84	

iFLC/niFLC ratios for N Latex FLC and Sebia FLC correlating with the Freelite iFLC/niFLC ratio ≥ 100 determination of Cohens Kappa revealed best concordances for N Latex at a threshold of ≥50 and for Sebia FLC at a threshold of ≥20 using Kappa statistics (Supplementary Fig. S1). Considering the iFLC/niFLC ratio > 20, we determined an equivalent ratio of >8 for both, N Latex FLC and Sebia FLC. Using these thresholds, adequate concordance between the assays was achieved (Table 3) and equivalent cutoff ratios for both, N Latex FLC and Sebia FLC, are proposed (Table 4).

**Table 4:** Proposed thresholds for equivalent iFLC/niFLC ratios between different FLC assays.

iFLC/niFLC ratio for	Freelite	N Latex FLC	Sebia FLC
MM requiring therapy	≥100*	≥50	≥20
High-risk SMM	>20*	>8	>8

Shown are iFLC/niFLC ratios of N Latex FLC and Sebia FLC equivalent to Freelite iFLC/niFLC ratio as part of diagnosis of MM requiring therapy and high-risk SMM

*according to references^8,10^

### Patient cases

**Fig. 3:**
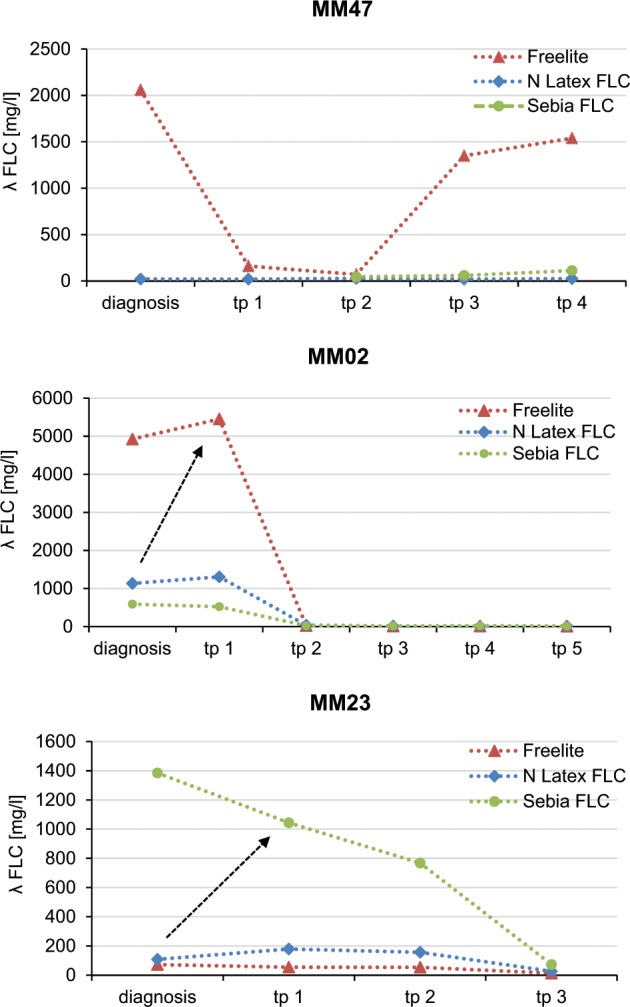
Discrepancies of light-chain values in the clinical course of treatment. Shown are λ FLC concentrations measured by Freelite, N Latex FLC, and Sebia FLC in clinical course of patients with clinically leading λ LCMM at indicated time points (tp). Patient MM47 represents a clinical course with partial response under initiated therapy resulting in a later documented progressive disease (measured by Freelite). Sebia FLC measurements, which were only accessible for time points 2–4, showed a markedly lower λ FLC. N Latex FLC results did not detect pathological λ FLC concentrations. Patient MM02 represents clinical stable disease after the first follow-up (measured by Freelite). λ FLC determination by Sebia FLC or N Latex FLC at diagnosis with response assessment with Freelite at tp1 might have lead to the wrong conclusion of progressive disease (indicated by arrow). Finding of markedly higher values can be observed also the other way around: in patient MM23, Sebia FLC detected markedly higher λ FLC values compared with Freelite and N Latex FLC.

Examples with marked discrepancies in the determination of FLC using different assays are shown in Supplementary Table 6 and Fig. 3. The first patient with λ LCMM (MM47) shows a clinical course with an initial partial response to initiated therapy, but subsequently showing progressive disease. These findings are based on Freelite FLC measurements. In contrast, N Latex FLC did neither detect pathological λ FLC concentrations at diagnosis nor in follow-up measurements. The results of Sebia FLC measurements, which were only accessible for time points 2–4, showed markedly lower λ FLC concentrations compared with Freelite.

The second patient (MM02) showed λ FLC concentrations of 4925, 591, and 1135 mg/l using Freelite, Sebia FLC, or N Latex FLC at diagnosis, respectively.

The third patient (MM23) shows that similar findings can be observed the other way around: in this case Sebia FLC detected markedly higher values of λ FLC (1385 mg/l at diagnosis) compared with Freelite (73 mg/l at diagnosis) and N Latex FLC (108 mg/l at diagnosis).

As shown, using different FLC assays, FLC values can be detected at different levels, and sometimes FLC evaluation is totally missed by one single test.

## Discussion

Determination of FLC has become standard of care in diagnosis and management of plasma cell disorders. Up to now, FLC analysis was mostly performed using the Freelite test based on polyclonal antibodies. With the introduction of the N Latex FLC assay, which is based on monoclonal antibodies, and just recently the introduction of a new assay based on polyclonal antibodies with ELISA detection (Sebia FLC), the landscape of FLC analysis had substantially changed. So far, only a few comparison analyses between N Latex FLC and Freelite^14–19^ and between Sebia FLC and Freelite^13,20,21^ exist, which have shown discrepancies in the determination of light-chain values and in κ/λ ratio. Here, we compare to the best of our knowledge for the first time all three available FLC assays in a single-center study.

We show overall higher total values for κ FLC and κ/λ ratio with Freelite compared with N Latex FLC with a strong correlation, however, higher variability for λ FLC. These results are in line with previously reported data^14,19^.

When comparing Sebia FLC assay to N Latex FLC and Freelite, Sebia FLC shows similar results compared with N Latex FLC with markedly lower values of κ and κ/λ ratio, when compared with Freelite. This again confirms previously published data as evaluated in the IFM and HOVON trials^20,21^, which, however, did not compare all three assays. In this analysis, concordance between all three assays for κ FLC was good, but only moderate for λ FLC. In contrast to κ FLC, λ FLC build more often dimeric and oligomeric complexes, which may contribute to the high variability in determination of λ FLC^26^. However, detailed reasons and underlying mechanisms are still unclear.

Despite strong correlation and good agreement rates, FLC assays cannot be used interchangeably. As shown in the patient examples, in clinical practice this may result in misinterpretation in response assessment and overall disease course.

In routine clinical practice, further challenges may occur. In oligosecretory MM, measurable disease is defined by iFLC ≥ 100 mg/l. Using N Latex FLC or Sebia FLC some patients might miss this definition because of absolute lower FLC values.

The determination of sCR requires a normal κ/λ ratio. Similarly then, the use of different assays may lead to different results.

In plasma cell dyscrasias other than MM, assessment of FLC is even more important and has potentially wider implications in treatment decisions. In AL-amyloidosis and LCDD, response evaluation is nearly solely based on percentage and absolute decrease of difference of involved to noninvolved FLC. Palladini et al. showed recently poor concordance of Freelite and N Latex FLC in discrimination of responses in AL-amyloid patients^27^. In this study, response to therapy was predicted by >33% decrease in N Latex FLC dFLC instead of >50% decrease in Freelite dFLC.

The clinical importance of FLC measurement was recently highlighted by the introduction of a distinct FLC ratio of involved FLC (iFLC)/noninvolved FLC (niFLC) for determination of high-risk SMM (iFLC/niFLC ratio > 20)^10^ and MM requiring treatment (iFLC/niFLC ratio ≥ 100)^8,9^. These recommendations were based on clinical trials conducted using the Freelite assay on a BN II nephelometer. Using these thresholds for FLC determination with N Latex FLC and Sebia FLC, low concordance rates with Freelite were observed. Two recently published investigations comparing either Freelite and N Latex FLC or Freelite and Sebia FLC, but not all three assays simultaneously, had recommended potential novel thresholds for iFLC/niFLC ratio ≥ 100 for N Latex FLC and Sebia FLC^14,20^. Bossuyt et al. compared diagnostic iFLC/niFLC ratio thresholds between Freelite and N Latex FLC using samples from MM and MGUS patients^14^. Kappa statistic was also used to establish novel FLC thresholds for N Latex FLC. Similar to our study, they found markedly reduced iFLC/niFLC ratios using N Latex FLC compared with Freelite. A threshold of ≥30 for N Latex FLC was proposed leading to an improved concordance with Freelite iFLC/niFLC ratio ≥ 100. However, Freelite measurements were performed on the Optilite system (The Binding Site). Proposed thresholds by the IWMG are based on measurements using the BN II nephelometer^9^ and transference of iFLC/niFLC ratios may not necessarily be given between different analyzers^28^.

Caillon et al. recently proposed a novel iFLC/niFLC ratio threshold for Sebia FLC^20^. They analyzed samples from SMM patients and used Freelite reagents on a BN II nephelometer and also calculated, in line with our results, a markedly lower threshold for Sebia FLC. This proposed threshold (iFLC/niFLC ≥ 16) was mathematically derived from Passing–Bablok regression analysis, and is quite similar to the proposed threshold in our study.

In summary, due to different patient cohorts, method of threshold calculation and different analyzers used in these studies, novel iFLC/niFLC ratio thresholds are hardly comparable. Our study is the first comparing all three available FLC assays simultaneously in a well-defined patient cohort. In addition, Freelite measurements were performed on the BN II nephelometer, which was used for the establishment of iFLC/niFLC thresholds recommended by the IMWG. Based on our findings, we here propose new thresholds for N Latex FLC and Sebia FLC for both thresholds, iFLC/niFLC ratio > 20 and iFLC/niFLC ratio ≥ 100 (Table 4) allowing adequate concordance between results of Freelite and of N Latex FLC and Sebia FLC.

However, this study has several limitations: our analysis is based on a single-center study with a limited number of patients. Most of the patients included in the study were newly diagnosed MM, and only a small number of patients had SMM. The proposed iFLC/niFLC ratios are mathematically calculated. Therefore, further clinical studies are needed to confirm these thresholds in larger cohorts. Furthermore, comparison of FLC assay results in a screening population remains to be seen.

In conclusion, based on our findings, the three different FLC assays should not be used interchangeably. We currently do not see an assay which should be preferably used or recommended, all three assays have limitations, advantages, and disadvantages. However, to assure correct classification and response assessment in primary diagnosis and during follow-up as well as to correctly interpret myeloma disease course, the use of assay-specific thresholds, ideally adapted by the IMWG, regarding FLC thresholds as well as the obligation to always state the used assay in all laboratory reports is crucial and should be consequently implemented in the clinical routine.

## Supplementary information

Supplemental Data

Supplemental Figure 1
