# Supplementary material for: Comparison of three different serum-free light-chain assays—implications on diagnostic and therapeutic monitoring of multiple myeloma
Source: Blood Cancer J. 2020 Jan 9;10(1):2. doi: 10.1038/s41408-019-0267-8 (PMC6949235; doi:10.1038/s41408-019-0267-8)
Supplement: Supplementary file 1 — Supplemental Data [file 41408_2019_267_MOESM1_ESM.docx]

**Supplemental Data**

**Supplemental methods: Determination of laboratory parameters**

Serum protein electrophoresis (SPE) and immunotyping were performed on capillary electrophoresis system (capillarys 2; Sebia, Evry, France) according to manufacturer’s instructions.

**Supplemental Figure 1:**

**Determination of equivalent iFLC/niFLC thresholds**

Kappa statistic was applied to calculate interobserver agreement between Freelite and N Latex FLC or Sebia FLC using samples from patients at the beginning of the study (N=42). **(A)** Interobserver agreement between Freelite iFLC/niFLC threshold ≥100 and N Latex FLC or Sebia FLC was calculated using thresholds for N Latex and Sebia FLC between 20 and 100. **(B)** Interobserver agreement between Freelite iFLC/niFLC threshold ≥20 and N Latex FLC and Sebia FLC was calculated using thresholds for N Latex and Sebia FLC between 4 and 20. Dotted lines indicate 95% confidence intervals.

**Supplemental Table 1: Reference ranges of FLC assays according to respective manufacturer**

|  | **κ FLC [mg/l]** | **λ FLC [mg/l]** | **κ/λ ratio** |
| --- | --- | --- | --- |
| **Freelite** | 3.30 – 19.40 | 5.71 – 26.30 | 0.26 – 1.65 |
| **N Latex FLC** | 6.7 – 22.4 | 8.3 – 27.0 | 0.31 – 1.56 |
| **Sebia FLC** | 5.2 – 15.3 | 8.2 – 18.1 | 0.37 – 1.44 |

**Supplemental Table 2: Linearity of κ and λ FLC determined by Sebia FLC.** Serum samples with elevated κ and λ FLC concentrations were manually diluted (1:2, 1:4, 1:8, 1:16, 1:32) using Sebia FLC dilution buffer and measured twice using Sebia FLC reagents. Shown are mean FLC concentrations [mg/l] and the corresponding linear regression equations.

| **Dilution** | **κ FLC** | **λ FLC** |
| --- | --- | --- |
| **-** | 41.41 | 38.07 |
| **1:2** | 27.72 | 21.31 |
| **1:4** | 13.04 | 12.71 |
| **1:8** | 7.58 | 8.99 |
| **1:16** | 4.13 | 4.46 |
| **1:32** | 2.16 | 3.23 |

κ FLC: y = 0.82x + 2.50 R^2^ = 0.97

λ FLC: y = 0.79x + 3.20 R^2^ = 0.99

**Supplemental Table 3: Intraassay precision of Sebia FLC.** Κ and λ FLC concentrations of four patient samples were measured in duplicates to determine intra-assay precision. Shown are values of κ and λ FLC concentrations [mg/l], the calculated mean, standard deviation (SD) and coefficient of variation (CV%) of each sample.

|  | **κ FLC low** | **κ FLC high** | **λ FLC low** | **λ FLC high** |
| --- | --- | --- | --- | --- |
| **1** | 6.2 | 24.9 | 3.7 | 26.0 |
| **2** | 5.6 | 30.1 | 3.8 | 28.8 |
| **3** | 5.0 | 28.4 | 3.3 | 32.7 |
| **4** | 5.7 | 27.3 | 4.6 | 26.4 |
| **5** | 6.1 | 27.6 | 4.6 | 26.7 |
| **6** | 5.2 | 27.9 | 5.0 | 27.2 |
| **7** | 5.9 | 26.2 | 4.4 | 26.0 |
| **8** | 5.1 | 29.0 | 4.2 | 32.0 |
| **9** | 5.3 | 25.0 | 3.0 | 31.8 |
| **10** | 5.5 | 26.9 | 3.7 | 33.2 |
| **Mean** | 5.6 | 27.3 | 4.0 | 29.1 |
| **SD** | 0.4 | 1.7 | 0.6 | 3.0 |
| **CV%** | 7.7% | 6.1% | 15.7% | 10.4% |

**Supplemental Table 4: Interassay precision of κ FLC using Sebia FLC.** Κappa (κ) FLC concentrations [mg/l] of five patient samples were measured in duplicate on ten different plates to determine intra-assay precision. Shown are values of κ FLC concentrations [mg/l] and the calculated mean, standard deviation (SD) and coefficient of variation (CV%) of each sample.

|  | **κ FLC 1** | **κ FLC 2** | **κ FLC 3** | **κ FLC 4** | **κ FLC 5** |
| --- | --- | --- | --- | --- | --- |
| **1** | 1.8 | 4.3 | 11.7 | 28.4 | 76.8 |
| **2** | 1.4 | 3.4 | 9.3 | 24.8 | 74.0 |
| **3** | 2.3 | 4.2 | 12.3 | 29.7 | 101.0 |
| **4** | 1.7 | 3.6 | 9.3 | 32.4 | 103.9 |
| **5** | 2.4 | 3.9 | 10.6 | 29.0 | 96.7 |
| **6** | 2.4 | 4.5 | 10.3 | 31.4 | 77.4 |
| **7** | 2.9 | 5.3 | 12.6 | 34.9 | 95.4 |
| **8** | 1.5 | 3.8 | 11.3 | 43.7 | 96.8 |
| **9** | 2.2 | 5.4 | 13.2 | 33.0 | 97.4 |
| **10** | 2.2 | 4.5 | 13.0 | 38.0 | 90.0 |
| **Mean** | **2.1** | **4.3** | **11.4** | **32.5** | **90.9** |
| **SD** | **0.5** | **0.7** | **1.4** | **5.4** | **10.9** |
| **CV%** | **23.1%** | **15.7%** | **12.7%** | **16.5%** | **12.0%** |

**Supplemental Table 5: Interassay precision of λ FLC using Sebia FLC.** Lambda (λ) FLC concentrations [mg/l] of five patient samples were measured in duplicate on ten different plates to determine intra-assay precision. Shown are values of λ FLC concentrations [mg/l] and the calculated mean, standard deviation (SD) and coefficient of variation (CV%) of each sample.

|  | **λ FLC 1** | **λ FLC 2** | **λ FLC 3** | **λ FLC 4** | **λ FLC 5** |
| --- | --- | --- | --- | --- | --- |
| **1** | 2.0 | 4.6 | 10.2 | 25.9 | 69.3 |
| **2** | 2.5 | 4.7 | 9.9 | 24.6 | 57.0 |
| **3** | 2.8 | 5.7 | 12.9 | 30.9 | 80.2 |
| **4** | 2.5 | 5.6 | 11.4 | 31.1 | 83.7 |
| **5** | 2.2 | 4.7 | 12.6 | 32.9 | 67.5 |
| **6** | 2.6 | 5.2 | 10.8 | 30.3 | 79.5 |
| **7** | 2.7 | 4.8 | 12.8 | 30.5 | 77.2 |
| **8** | 2.6 | 6.5 | 11.1 | 31.2 | 92.6 |
| **9** | 2.7 | 5.2 | 11.8 | 32.9 | 74.8 |
| **10** | 2.6 | 6.2 | 11.9 | 33.9 | 78.8 |
| **Mean** | **2.5** | **5.3** | **11.5** | **30.4** | **76.1** |
| **SD** | **0.2** | **0.7** | **1.1** | **3.0** | **9.7** |
| **CV%** | **9.2%** | **12.5%** | **9.2%** | **9.9%** | **12.8%** |

**Supplemental Table 6: Patient cases**

Shown are κ and λ FLC values and the corresponding iFLC/niFLC ratio of three patients with marked discrepancies in the determination of FLC.

| **Patient** | **tp** | **κ light chain [mg/l]** | | | **λ light chain [mg/l]** | | | **iFLC/niFLC ratio** | | |
| --- | --- | --- | --- | --- | --- | --- | --- | --- | --- | --- |
|  |  | **Freelite** | **N Latex FLC** | **Sebia FLC** | **Freelite** | **N Latex FLC** | **Sebia FLC** | **Freelite** | **N Latex FLC** | **Sebia FLC** |
| **MM02** | b | 7.8 | 8.3 | 8.2 | 4925.2 | 1135.7 | 591.1 | 629.7 | 137.3 | 72.1 |
|  | 1 | 7.0 | 7.4 | 7.5 | 5836.5 | 1136.5 | 512.0 | 833.8 | 153.6 | 68.7 |
|  | 2 | 2.0 | 6.7 | 7.8 | 5452.6 | 1308.3 | 524.4 | 2662.7 | 195.3 | 67.3 |
|  | 3 | 15.2 | 12.0 | 11.6 | 28.2 | 36.8 | 25.6 | 1.9 | 3.1 | 2.2 |
|  | 4 | 13.5 | 13.6 | 9.7 | 14.8 | 15.8 | 17.7 | 0.9 | 0.9 | 0.5 |
| **MM23** | b | 0.3 | 1.4 | 1.4 | 72.7 | 108.5 | 1385.0 | 215.7 | 78.7 | 957.4 |
|  | 1 | 0.1 | 1.2 | 2.3 | 54.9 | 179.2 | 1044.0 | 914.9 | 149.3 | 463.5 |
|  | 2 | 0.3 | 1.3 | 4.1 | 53.9 | 157.0 | 767.8 | 168.4 | 120.8 | 186.9 |
|  | 3 | 2.4 | 3.8 | 3.2 | 13.9 | 26.3 | 73.1 | 5.8 | 6.9 | 22.6 |
| **MM47** | b | 9.0* | 9.0* | N/A | 2060.0* | 21.8* | N/A | 219.0* | 3.0* | N/A |
|  | 1 | 17.0* | 22.0* | N/A | 161.0* | 20.1* | N/A | 9.0* | 1.0* | N/A |
|  | 2 | 49.8 | 36.2 | 30.4 | 70.4 | 27.9 | 45.3 | 1.4 | 0.8 | 1.5 |
|  | 3 | 19.6 | 12.8 | 4.4 | 1350.0 | 19.3 | 60.1 | 68.9 | 1.5 | 13.7 |
|  | 4 | 12.7 | 9.9 | 15.2 | 1540.0 | 25.8 | 112.8 | 121.3 | 2.6 | 7.4 |

tp = timepoint, b = baseline, κ = kappa, λ = lambda, iFLC/niFLC = involved free light chain/ non-involved free light chain, FLC = free light chain, N/A = not applicable.

*results of FLC determination at baseline and time point 1 were not included in the study due to missing values by Sebia FLC.
