# Supplementary figures and images for: Comparison of three different serum-free light-chain assays—implications on diagnostic and therapeutic monitoring of multiple myeloma
Source: Blood Cancer J. 2020 Jan 9;10(1):2. doi: 10.1038/s41408-019-0267-8 (PMC6949235; doi:10.1038/s41408-019-0267-8)

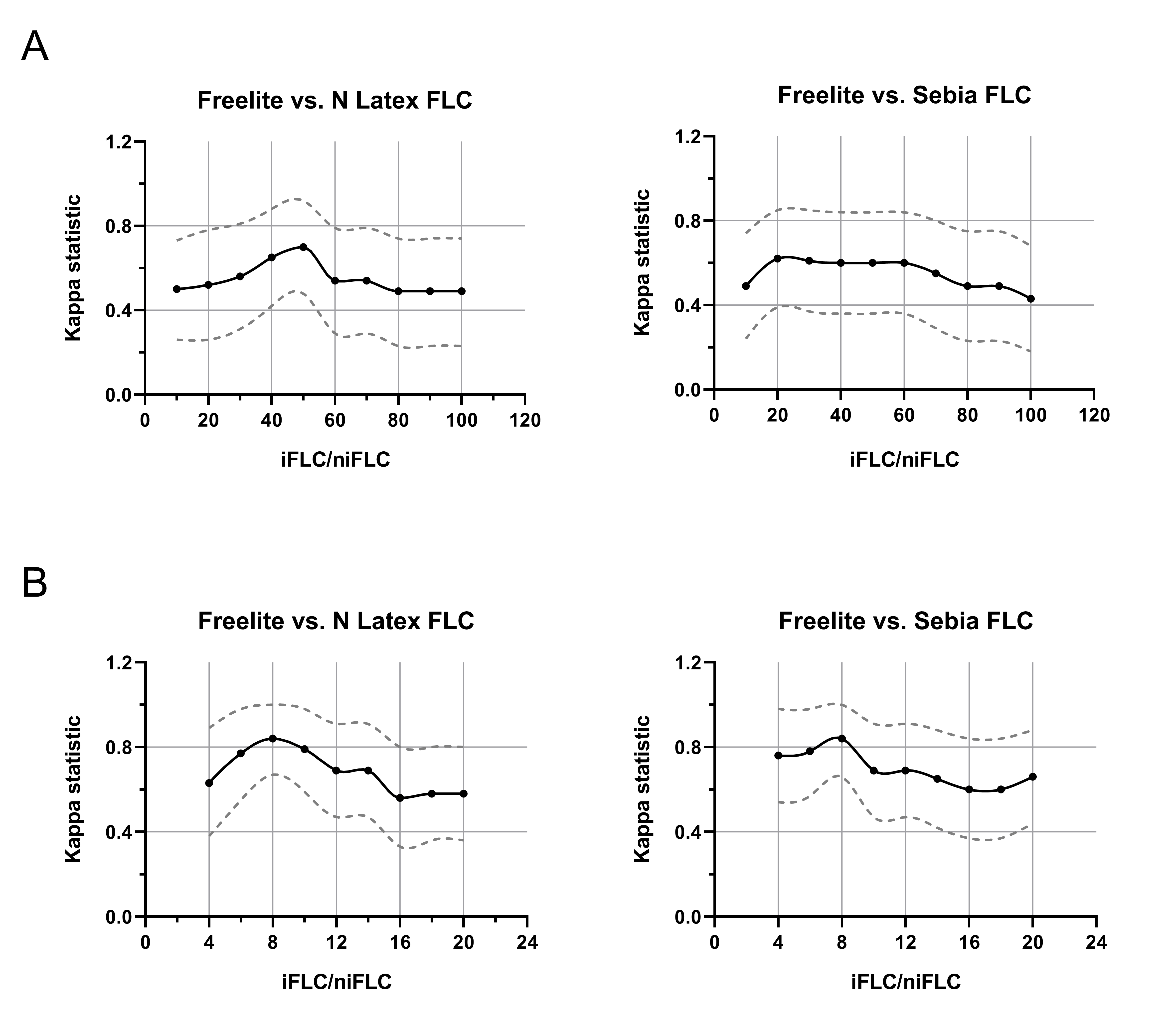

Supplement: Supplementary file 2 — Supplemental Figure 1 [file 41408_2019_267_MOESM2_ESM.tif]
